# Data of the immersion enthalpy of activated carbon in benzene and cyclohexane. Influence of the content of surface oxygenated groups

**DOI:** 10.1016/j.dib.2018.11.137

**Published:** 2018-11-30

**Authors:** Diana Hernández-Monje, Liliana Giraldo, Juan Carlos Moreno-Piraján

**Affiliations:** a Departamento de Química, Facultad de Ciencias, Universidad Nacional de Colombia, Sede Bogotá, Carrera 30 No. 45-03, Bogotá, Colombia; b Departamento de Química, Facultad de Ciencias, Universidad de los Andes, Carrera 1 este No. 18A-12, Bogotá, Colombia

## Abstract

The objective of this data article is to show by calorimetric curves and the immersion enthalpies the differences between the interactions that occur when two activated carbons of different textural and chemical properties are put in contact with C_6_ compounds (an aromatic and a closed chain aliphatic: benzene and cyclohexane, respectively) in their pure state, and subsequently in mixtures thereof, with different molar composition. The greatest interaction occurs with the activated carbon that has the lower content of oxygen groups on the surface, both for the pure solvents, as for the mixtures; As for wetting liquids, there is a greater interaction with benzene (−∆H_im_: 94.98–106.40 J g^−1^) than with cyclohexane (−∆H_im_: 21.23–65.97 J g^−1^). The immersion enthalpy values for the different molar fractions are between −36.51 and −79.69 J g^−1^ for the oxidized sample, and between −50.43 and −85.59 J g^−1^ for the sample without chemical modification.

**Specifications table**

**Table:** 

Subject area	Physical chemistry
More specific subject area	Thermodynamic
Type of data	Table, figure
How data were acquired	Immersion Calorimetry
Data format	Raw, analyzed, etc
Experimental factors	A raw activated carbon that was prepared from coconut shell by physical activation (*GAC*), and an activated carbon oxidized with nitric acid 6 M solution (*OAC*) were used.
Experimental features	Activated carbons were immersed in pure solvents (benzene and cyclohexane) and into mixtures of benzene-cyclohexane. To determine the enthalpy of immersion, the solvent or the mixture were placed in a cell assembled to the heat reservoir of the calorimeter; then, the activated carbon was placed in a glass vial also fitted to the calorimeter cell. Next, the immersion of the sample into the liquid was performed and the electric calibration was executed.
Data source location	Universidad Nacional de Colombia, Chemistry Department, Bogotá, Colombia.
Data accessibility	Data are provided in this article
Related research article	Diana Hernández-Monje, Liliana Giraldo and Juan Carlos Moreno-Piraján
	*Data of the immersion enthalpy of activated carbon in benzene and cyclohexane. Influence of the content of surface oxygenated groups*
	Data correspond to calorimetric curves derived from the immersion of two different activated carbons into C_6_ solvents (aromatic and closed chain aliphatic compounds), mixtures of them and their corresponding immersion enthalpy values.

**Value of the data**

• This data article shows the interactions between two C_6_ compounds that differ in their arrangement, molecular size and other physicochemical properties with activated carbons with different textural and chemical properties.
• The data want to show how the change in the composition of the benzene–cyclohexane mixture and the properties of adsorbates affect the adsorbent–adsorbate and solute–solvent interaction.
• These data present binary mixtures of the adsorbates to different molar fractions, increasing the concentration of the solute to go from the enthalpy of immersion of the pure solvent (cyclohexane), to the enthalpy of the pure solute (benzene); showing how the addition of another component with aromatic structure similar to that found in activated carbon affects the interaction cyclohexane – activated carbon.
• The intensity of the interactions can be evaluated by testing another type of solids and C_6_ molecules, and compare the results.

## 1 Data

These data correspond to calorimetric curves obtained by immersion calorimetry and their corresponding immersion enthalpy values for two activated carbons: a raw one that does not present modifications (*GAC*) and another submitted to an oxidation process with HNO_3_ 6 M solution (*OAC*), into two pure solvents C_6_ (benzene and cyclohexane) and mixtures of different molar fraction of benzene in cyclohexane (0.2, 0.4, 0.6, 0.8). Table 1 shows the values of enthalpy of immersion of *GAC* and *OAC* into the pure liquids (benzene and cyclohexane) and the mixtures with concentrations between 0.2 and 0.8 M fraction.

**Table 1:** Values of enthalpy of immersion of GAC and OAC into the pure liquids (benzene and cyclohexane) and the mixtures with concentrations between 0.2 and 0.8 M fraction.

Sample	−∆H_im_	−∆H_im_	−∆H_im_ (0.2^a^)	−∆H_im_ (0.4^a^)	−∆H_im_ (0.6^a^)	−∆H_im_ (0.8^a^)
	C_6_H_6_	C_6_H_12_	C_6_H_6_–C_6_H_12_	C_6_H_6_–C_6_H_12_	C_6_H_6_–C_6_H_12_	C_6_H_6_–C_6_H_12_
	(J g^−1^)	(J g^−1^)	(J g^−1^)	(J g^−1^)	(J g^−1^)	(J g^−1^)
GAC	106.40	65.97	50.43	55.47	90.49	85.59
OAC	94.98	21.23	36.51	43.11	79.69	57.50

a It corresponds to the mixture of benzene in cyclohexane at those molar fractions.

## 2 Experimental design, materials and methods

### 2.1 Activated carbon

Two samples of activated carbon were used. The raw was prepared from coconut shell by physical activation (*GAC*), it was sieved to a particle size of 1 mm, and it was washed with distilled water, then it was dried for 24 h at 363 K and it was stored in containers under nitrogen atmosphere. Then, a chemical modification of the raw carbon was performed (treatment of oxidation with HNO_3_ 6 M solution, in an inert atmosphere) for the development of oxygenated surface groups (*OAC*).

### 2.2 Enthalpy of immersion of activated carbon in solvents and mixtures

Activated carbons were immersed in pure solvents (benzene and cyclohexane – Merck-brand analytical reagents) and into mixtures of benzene–cyclohexane with concentrations between 0.2 and 0.8 M fraction. To determine the enthalpy of immersion, 10 ml of the solvent were placed in a stainless steel cell assembled to the heat reservoir of the calorimeter at 298 K; then, 100 mg of activated carbon were placed in a glass vial fitted to the calorimeter cell. Next, the capture of the output electric potential started and the stabilization of the calorimeter until base line was achieved; later, the immersion of the sample into the liquid was performed and the resulting thermal changes were recorded until the baseline was attained again. Finally, a post-period was recorded, and the electric calibration was executed [1], [2], [3], [4].

**Fig. 1:**
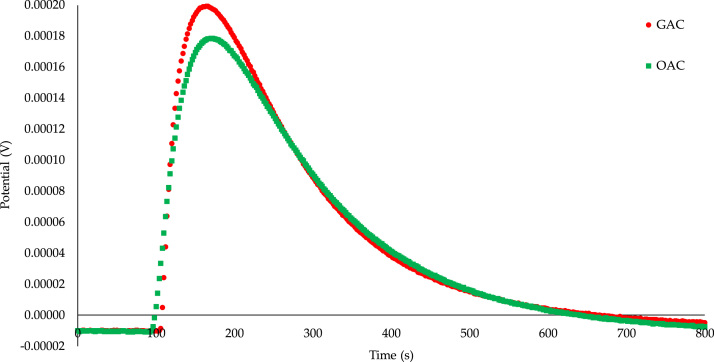
Calorimetric curves of the immersion of *GAC* and *OAC* into benzene.

**Fig. 2:**
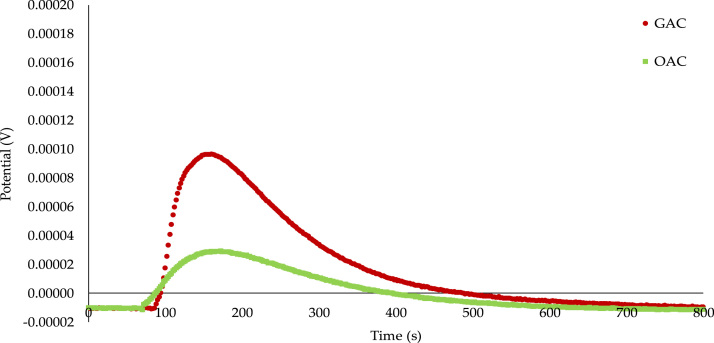
Calorimetric curves of the immersion of *GAC* and *OAC* into cyclohexane.

**Fig. 3:**
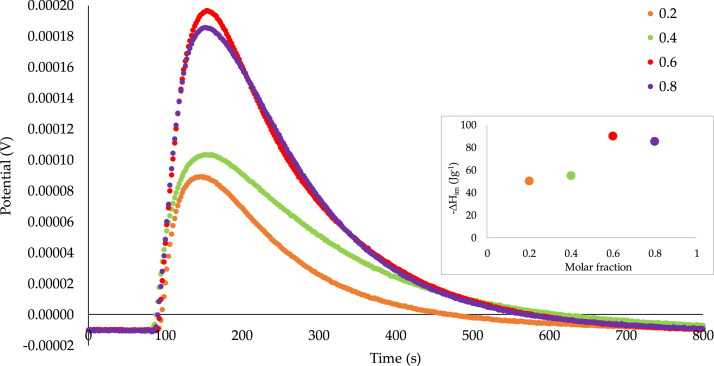
Calorimetric curves of the immersion of *GAC* into a mixture of benzene–cyclohexane with concentrations between 0.2 and 0.8 M fraction.

**Fig. 4:**
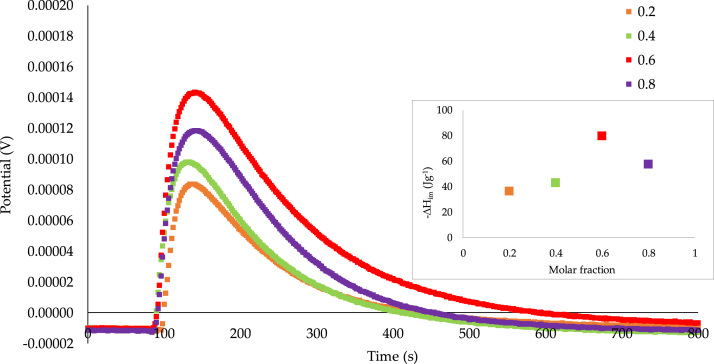
Calorimetric curves of the immersion of *OAC* into a mixture of benzene–cyclohexane with concentrations between 0.2 and 0.8 M fraction.

**Fig. 5:**
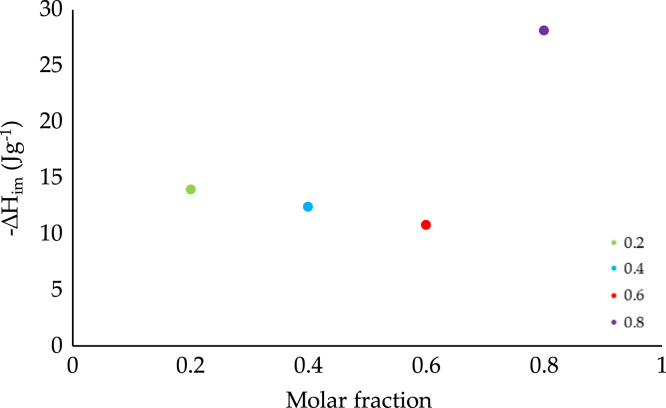
Difference between the enthalpy of immersion of *GAC* and *OAC* into the mixture of benzene–cyclohexane as a function of the molar fraction.

A plot of the variation of the electrical potential versus time could be used for the calculation of immersion enthalpy (Table 1); the plot contains two peaks: the first corresponds to the immersion process, breaking the cell and wet sample, and the second to electrical calibration of the calorimeter. In this work only the peak for the immersion process is shown due to the second is used to determine the calorimeter constant, not the interaction between the solid and the adsorbate (Fig. 1, Fig. 2, Fig. 3, Fig. 4, Fig. 5) [5].

The electrical calibration of the equipment is carried out by heating the system with a resistance of 100 Ω that allows to calculate the electrical work dissipated in the system, taking into account that:

(1) $$W_{elec}=Potential(V)*Current(A)*Time(s)$$

when obtaining W_elec_, the calorimeter constant (K) is calculated, which corresponds to:

(2) $$K=\frac{W_{elec}}{area\;under\;the\;curve\;of\;the\;calibration\;peak}$$

once the calorimeter constant is determined, the energy of immersion (J) is calculated:

(3) $$E_{im}=\frac{K}{area\;under\;the\;curve\;of\;the\;immersion\;peak}$$

and then, the immersion enthalpy (J g^−1^) in function of the mass of solid is obtained:

(4) $$H_{im}=\frac{E_{im}}{mass\;of\;the\;activated\;carbon}$$

Fig. 1 shows the calorimetric curves of the immersion of *GAC* and *OAC* into benzene. Since the area under the curve is directly proportional to the intensity of the interaction, a higher interaction is evidenced for the raw carbon with benzene than for the one with the greater number of oxygenated groups [6]. The enthalpies of immersion of the solids in benzene are GAC: −106.40 J g^−1^; OAC: −94.98 J g^−1^).

Fig. 2 shows the calorimetry curves of the immersion of *GAC* and *OAC* into cyclohexane. As in Fig. 1, it also shows a higher interaction for the raw carbon with the liquid than for the oxidized one, but the values with respect to benzene, are lower.

In order to show if there are changes in the interactions with the solid when the concentration of the solute is changed, calorimetric determinations were made at different molar concentrations (0.2, 0.4, 0.6, 0.8) for the two solids. In Fig. 3, for *GAC*, the calorimetric curves show that as the solute increases, the interaction increases (0.2, −50.43, 0.4 and −55.47 J g^−1^), reaching a maximum in 0.6 (−90.49 J g^−1^); however, when there is already a greater amount of benzene with respect to cyclohexane, the interaction decreases (0.8: -85.59 J g^−1^).

The same behavior of Fig. 3 is observed in Fig. 4 for *OAC*; however, the intensity of the interaction is lower for the sample with the greater number of oxygenated groups. Again, the potential got higher as the solute increased (0.2, −36.51, 0.4 and −43.11 J g^−1^), reaching a maximum in 0.6 (−79.69 J g^−1^); finally, when there is already a greater amount of benzene with respect to cyclohexane, the interaction decreases (0.8 and −57.50 J g^−1^).

According to above, the higher values of immersion enthalpies for the mixtures correspond to a concentration of 0.6 M fraction (*GAC*: −90.49 J g^−1^; *OAC*: −79.69 J g^−1^). Again, there is a greater interaction with the raw activated carbon than with the oxidized one.

To determine the influence of the presence of surface oxygenated groups in the interaction between the activated carbon and the mixture, the difference between the values of immersion enthalpy of the sample without chemical modification (*GAC*) and the one that was chemically modified with nitric acid (*OAC*) was calculated. These values were plotted as a function of molar fraction, and they are shown in Fig. 5. The difference decreases as the concentration increases (0.2–0.6); however, in the maximum concentration of benzene in cyclohexane (0.8), the difference between the two samples increases significantly.
