# Supplementary material for: Data of the immersion enthalpy of activated carbon in benzene and cyclohexane. Influence of the content of surface oxygenated groups
Source: Data Brief. 2018 Nov 30;22:83–9. doi: 10.1016/j.dib.2018.11.137 (PMC6297062; doi:10.1016/j.dib.2018.11.137)
Supplement: Supplementary file 1 — Supplementary material [file mmc1.doc]

Nov 20th, 2018

Dears,

**Data in Brief,**

We wish to submit an original research data entitled “***Determination of immersion the enthalpy of activated carbon in benzene and cyclohexane. Influence of the content of surface oxygenated groups***” for consideration in Data in Brief. We confirm that this work is original and has not been published elsewhere, nor is it currently under consideration for publication elsewhere.

In this research, the objective of this article is to show by the immersion calorimetry and the determination of the enthalpies of immersion, the differences between the interactions that occur when putting in contact two activated carbons of different textural and chemical properties with C_6_ compounds (an aromatic and a closed chain aliphatic: benzene and cyclohexane, respectively) in their pure state, and subsequently in mixtures thereof, with different molar composition. The greatest interaction occurs with the activated carbon with lower content of oxygen groups on the surface, both for the pure solvents, as for the mixtures; As for wetting liquids, there is a greater interaction with benzene (-∆H_im_: 94.98 - 106.40 Jg^-1^) than with cyclohexane (-∆H_im_: 21.23 - 65.97 Jg^-1^). The immersion enthalpy values ​​for the different molar fraction are between -36.51 and -79.69 Jg^-1^ for the oxidized sample, and between -50.43 and -85.59 Jg^-1^ for the sample without chemical modification.

We believe that this manuscript is appropriate for publication by the Data in Brief. We have no conflicts of interest to disclose. If you feel that the manuscript is appropriate for your journal.

Please address all correspondence concerning this manuscript to me at jumoreno@uniandes.edu.co. Thank you for your consideration of this manuscript.

Best regards,

Prof. Juan Carlos Moreno., PhD

Full Professor

University of the Andes

Bogotá, Colombia
